# microRNA‐193a‐5p Suppresses the Migratory Ability of Human KATO III Gastric Cancer Cells through Inhibition of Vimentin and MMP-9

**DOI:** 10.34172/apb.2022.018

**Published:** 2020-10-19

**Authors:** Amir Baghbanzadeh, Elham Baghbani, Khalil Hajiasgharzadeh, Saeed Noorolyai, Vahid Khaze, Behzad Mansoori, Masoud Shirmohamadi, Behzad Baradaran, Ahad Mokhtarzadeh

**Affiliations:** ^1^Immunology Research Center, Tabriz University of Medical Sciences, Tabriz, Iran.; ^2^Liver and Gastrointestinal Diseases Research Center, Tabriz University of Medical Sciences, Tabriz, Iran.; ^3^Department of Immunology, Tabriz University of Medical Sciences, Tabriz, Iran.

**Keywords:** miRNA-193a‐5p, Gastric Cancer, Gene Therapy, Apoptosis, Migration Assay

## Abstract

***Purpose:*** *microRNA-193a-5p* is one of the well-known tumor suppressor miRNAs in the body but in many cases, its expression became reduced in patients suffering from gastric cancer (GC). The main purpose of this study was to restore the function of this miRNA in human GC cells and investigating the effects of enhanced expression of *miR-193a-5p* on proliferation, apoptosis, and migration of GC cells upon in vitro transfection.

***Methods:*** The KATO III GC cells were treated with 100 nM of *miR-193a-5p* or negative control sequences. Following that, the MTT assay, flow cytometry assay, and wound-healing assay were applied to estimate the impacts of enhanced expression of this miRNA on the viability, apoptosis, and migration rate of the cells, respectively. Moreover, the total RNA was isolated and alterations in the mRNA expression ratio of migratory genes were measured by qRT-PCR techniques.

***Results:*** The findings designated that enhanced expression of *miR-193a-5p* suppressed the migratory ability of the cells, but had no significant effects on cell survival or apoptosis of the transfected cells. In addition, this inhibitory function of *miR-193a-5p* on the migration rate of the KATO III cell line occurs with concurrent suppression of vimentin and MMP-9 gene expression.

***Conclusion:*** It can be concluded that *miR-193a-5p* negatively influences the migratory ability of the cancerous cells and restoring its effects can be regarded as a promising target of future therapeutic interventions, especially for GC metastasis.

## Introduction

Gastric cancer (GC) is one of the most frequent types of cancers in the world that leads to high rates of cancer-related mortality each year.^1^ A recent analysis of GC patients’ statistics revealed that the incidence rate of this malignancy is gradually increasing in young populations especially in developing countries.^2^ Besides, the poor prognosis of GC patients after standard chemotherapy or radiotherapies along with inefficiency and adverse side effects of such existing therapies, emphasizes the urgent necessity for the development of new alternative GC treatment options.^3^ This disease has no distinct symptoms during its initial and non-metastatic stages, which leads to delayed diagnosis and the beginning of the treatment of the disease.^4^ In this cancer, with almost half of the cases, the liver is the most prevalent place for GC metastasis to occur, which is subsequently associated with a high mortality rate.^5^ Despite the progress in clinical innovations and the development of novel detection methods, most of the GC subjects are diagnosed in late stages with metastasis capacity.^6^ Hence, identifying the causes of metastasis occurrence and developing innovative therapeutic approaches to suppress cancer cell movement and migration and reversing the disease state to the normal level is of particular priority.^7^

GC is a result of the dysregulation of a combination of multiple factors including *Helicobacter pylori* infection, chronic inflammation, genetic susceptibility, chromosomal insufficiency, microsatellite instability, genetic polymorphisms as well as bad eating habits.^8^ In addition to these factors, the changes in the microRNA (miRNA) profile that extremely influence the expression of the downstream genes have been reported in many GC patients.^9^ MiRNAs are small non-coding RNAs, which about 1/3 of the human protein-coding genes could be under the regulation of these miRNAs.^10^ The miRNA machinery began by a non-perfect pairing of these nucleotides to the targeted mRNA, which leads to the subsequent formation of RISC complex and involvement of other relating mRNA degradation systems.^11^ Among these miRNAs, the impaired and unregulated expression of *miR-193a*family in numerous cancers is reported in several investigations.^12^ There has been increasing evidence that indicates their pivotal roles in cancer pathways.^13,14^ In the process of *miR-193a-3p*generation, the *pre-miR-193a*generates both *miR-193a-3p* and *miR-193a-5p*, based on the arm that is processed during their formation and consequently sets distinct targets for each of them.^15^ Similar to other tumor suppressor miRNAs, it became clear that the expression of *miRNA-193a-5p* in cancer samples is lower than those in normal adjuvant samples. In this context, the downregulated *miRNA-193a-5p* expression was reported in lung tumors,^16^ colorectal cancers,^13^ malignant melanomas,^17^ oral cancers,^18^ and acute myelogenous leukemia.^19^ Therefore, restoring the function of this miRNA as a well-known tumor suppressor may provide clinical significance.

Thus, we hypothesized that the dysregulation of* miRNA-193a-5p* may effectively affect GC cell properties such as deregulated migration signaling pathways, which leading to gastric tumor invasion and metastasis. To date, the exact impacts of *miR-193a-5p* in GC initiation and metastasis remains not completely understood. Altogether, because the degenerated expression of tumor suppressor miRNAs is greatly concerned in GC, in the current study, we tried to evaluate the effects of *miR-193a-5p* mimics on proliferation, apoptosis, and migration of the cells and investigate the expression of vimentin and MMP-9 genes in KATO III cell lines. *miR-193a-5p* may be a new target for the design of targeted therapy and may provide a potential biomarker to early detection and GC therapy.

## Materials and Methods

### Cell culture

The human GC cell lines AGS, MKN-45, and KATO III were received from Pasteur Institute of Iran and cultured in RPMI medium with 10% fetal bovine serum (FBS) (Gibco, USA) and 100 IU/mL penicillin/100 μg/mL streptomycin mixtures. The cultures were preserved at a 37°C incubator (Memmert, Schwabach, Germany) in a 95% humidified atmosphere of 5% CO_2_ and were used in the logarithmic phase of growth according to our previous studies.^20^ All of the assays were independently repeated three times.

### RNA preparation, cDNA synthesis, and qRT-PCR

The expression of *miR-193a-5p* and alterations in the expression of vimentin, Rock, c-Myc, and MMP-9 genes as putative targets of this miRNA were quantified by qRT-PCR. In brief, the cells from three different GC cell lines including AGS, MKN-45, and KATO III were cultured in 6 well plates at the density of 4×10^5^ cells per well. Afterward, total RNA was isolated by the TRIzol (RiboEx) and then, 1 μg of the extracted mRNA was utilized for cDNA synthesis using a kit (Biofact, South Korea). Following that, the qRT-PCR was conducted utilizing light cycler 96 (Roche Diagnostics, Mannheim, Germany). The data were analyzed using 2-^∆∆CT^ method. U6 and β-actin were served as internal parameters of miRNA and housekeeping controls for target genes, respectively. The sequences of each primer for the analyzed genes are listed in Table 1.

**Table 1:** Primer sets used for quantification of mRNA expression of target genes

**Genes**		**Sequences**
MMP-9	Forward	5’-ATTTCTGCCAGGACCGCTTCTAC-3’
	Reverse	5’-ATCCGGCAAACTGGCTCCTTC-3’
Vimentin	Forward	5’-AATCGTGTGGGATGCTACCT-3’
	Reverse	5’-CAGGCAAAGCAGGAGTCCA-3’
β-Actin	Forward	5’-TCCCTGGAGAAGAGCTACG-3’
	Reverse	5’-GTAGTTTCGTGGATGCCACA-3’
U6	Forward	5’-CTTCGGCAGCACATATACTAAAATTGG-3’
	Reverse	5’-TCATCCTTGCGCAGGGG-3’

### Transfection of miRNA

After the initial determination of the *miR-193a-5p* expression ratio in all three cells, the cell line with the lowest expression ratio was selected for the rest of the study. The *hsa-miR-193a-5p* sequences (5’-UCAUCUCGCCCGCAAAGACCCA-3’) and negative control miRNA (miR-NC) sequences were purchased from Microcynth (AG, Switzerland). Then, the selected cell line was cultured in an antibiotic-free medium in six-well plates at the density of 3×10^5^ cells and was transfected at about 80 percent confluency with diverse concentrations of miRNA mimic (50 nM, 75 nM, and 100 nM) with the jetPEI reagent (PolyPlus, France), according to the given transfection guidelines.^21^ Among these miRNA concentrations, the concentration that causes the greatest increase in *miR-193a-5p* expression (i.e. 100 nM) was selected for the following studies. After 6 h incubation in a cell culture incubator, RPMI which supplied with 20% FBS was added, and the cells kept for an additional 48 h prior to the beginning of the MTT, wound-healing, and qRT-PCR assays.

### MTT cell viability assay

The influences of *miR-193a-5p* transfection on the viability of the KATO III cells were assessed by MTT assay. Briefly, to this cytotoxicity measurement, approximately 15×10^3^ cells per well were cultured in 96-well plates and kept for 24 hours in the standard incubator. Following that, the cells were treated by 100 nM of *miR-193a-5p*mimic, which was the optimal concentration of miRNA and negative control miRNA (miR-NC) for 48 h at 37°C and 5% CO_2_ level. Following 48 hours, the medium was discarded and incubated with 2mg/mL of MTT (Sigma, Germany) and were kept for further 4 hours at 37°C incubator. Then, 200 mL of dimethyl sulfoxide (DMSO) was used to solubilize the resulting formazan crystals. After incubation at 37°C for 30 minutes, absorbance was recognized at wavelength 570 nm employing a SunriseTM microplate reader (Tecan, Switzerland).

### Apoptosis assay

To discover the modifications of apoptosis after *miR-193a-5p* mimic transfection, the apoptosis of the cells was assessed by flow cytometry (FCM) assay using an Annexin V/PI double staining kit (EXBIO, Czech Republic). To estimate the percentage of apoptosis of the cells, they were cultured at a seeded of 2×10^5^ cells in six-well plates. Next, wells were divided into two groups as miR-NC treated and transfected by miR-193a-5p mimic wells. After 48 h, the cells were stained, and then these stained cells were determined by an FCM instrument (FACSQuant; Milteny Biotec, Germany). The rate of apoptotic cells was measured and obtained data were investigated using FlowJo software (Treestar, Inc., San Carlos, CA).

### Cell migration assay

Wound healing assay (Scratch) was measured the impacts of *miR-193a-5p* mimic transfection on the migration rate of KATO III cells. For this analysis, 2×10^5^ of KATO III cells were seeded in the 24-well plates for 24 hours to reach the right confluency. Before transfection, we created a wound gap in the bottom of the plate using the tip of a yellow micropipette. After the removal of cell debris, the wells were classified into 2 groups (a treated group with 100 nM of *miR-193a-5p* mimic and the control miRNA groups). The plates were incubated for 48 hours at the standard incubator. During this period, the cells were monitored and photographed at 0, 24, and 48 hours after treatment. The migratory ability of the cells was assessed by estimating the gap between the edges of the wound by using ImageJ software.

### Statistical analysis

All data are shown as the mean ± SEM. GraphPad Prism 6 software (San Diego, CA, USA) was applied for statistical analysis. One-way analyses of variance were done to demonstrate statistical differences among groups, followed by Tukey test. The *P* values smaller than 0.05 were considered statistically significant.

## Results

### miR-193a-5p was downregulated in GC cell lines

**Figure 1:**
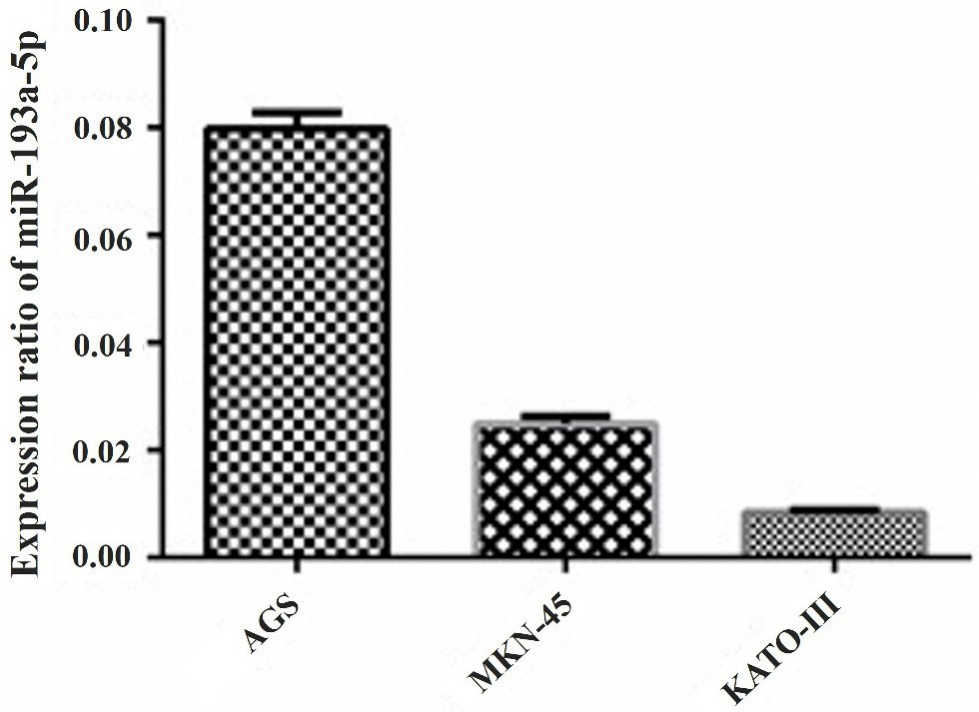
The qRT-PCR results revealed that *miR-193a-5p* has low expression levels in three GC cell lines including AGS, MKN-45, and KATO III cells. Comparably, the KATO III cell line had the lowest expression ratio of this miRNA in comparison with AGS and MKN-45.

The relative expression of *miR-193a-5p* assessed in three cell lines (AGS, MKN-45, and KATO III) was assessed (Figure 1), and the results revealed that this miRNA has low expression levels in all cell lines. Comparably, the KATO III cell line had the highest decrease in *miR-193a-5p* in comparison with AGS and MKN-45. Therefore, the highly metastatic KATO III cells were selected for the rest of the experiments.

### miR-193a-5p was upregulated following the transfection of the KATO III GC cells

**Figure 2:**
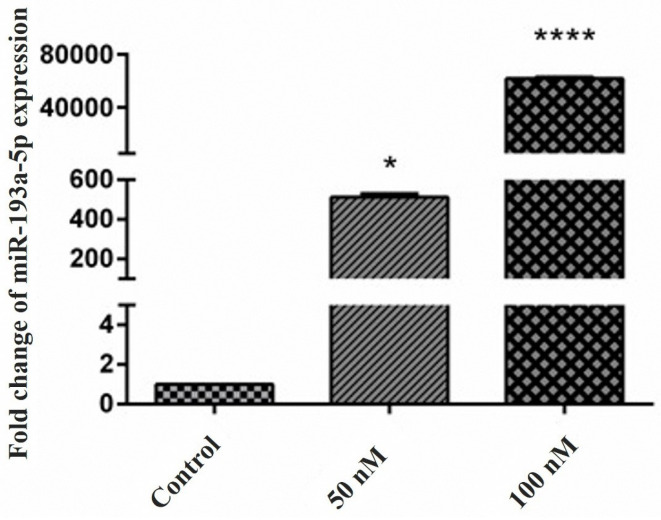
Restoration of *miR-193a-5p* expression was confirmed by the qRT-PCR. The up-regulation of *miR-193a-5p* in the transfected cells confirmed a successful miRNA transfection. High fold change increase in *miR-193a-5p* expression after 50 nM (*P < 0.05) and 100 nM (****P < 0.0001) was evident in transfected cells.

The findings indicated that the *miR-193a-5p* was downregulated in the KATO III cell line. According to these results, *miR-193a-5p* mimic transfection was performed for 24, 48, and 72 hours, and the best upregulation time was recognized at 48 hours (Data not shown). For optimal dose adjustment, the GC cells were transfected with two different doses of *miR-193a-5p* mimic; 50 nM (*P*< 0.05), and 100 nM (*P* < 0.0001) (Figure 2). According to these results, 100 nM of *miR-193a-5p* mimic was selected as the optimal concentration for all subsequent experiments.

### Transfection of miR-193a-5p had no significant effect on cell viability and apoptosis of KATO III cell line

**Figure 3:**
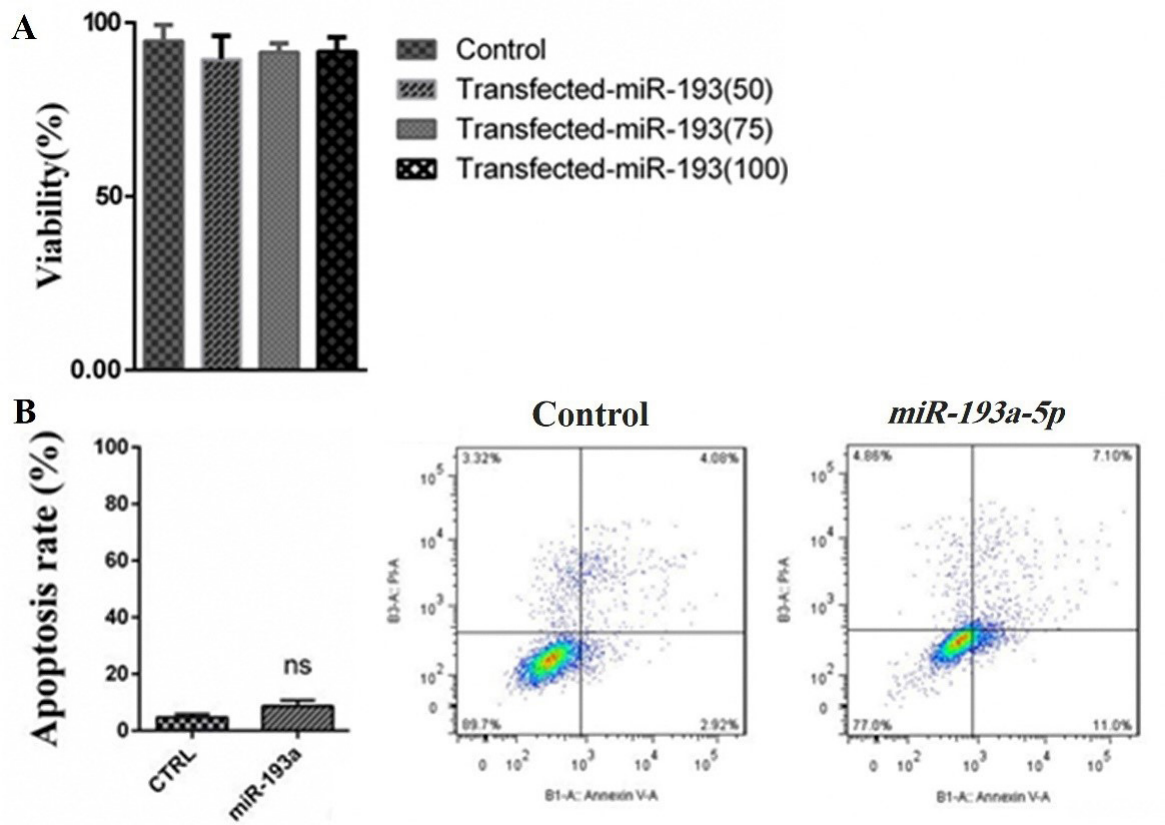
(A) The effects of *miR-193a-5p* transfection on KATO III cells viability. The results designated that the viability of the transfected cells with 50 nM, 75 nM, and 100 nM of *miR-193a-5p* mimics didn’t change in comparison to the control group. (B) To further evaluation of the possible effects of *miR-193a-5p* transfection on the apoptosis rate of KATO III cells, an FCM assay was conducted and the results showed that *miR-193a-5p* mimic had no significant impact on apoptosis occurrence in the cells compared to control cells.

To discover the consequences of *miR-193a-5pmimic*transfection, the MTT test was done to identify the effects of this transfection on the cell viability of KATO III cells. As presented in Figure 3A, enhanced expression of *miR-193a-5p* did not affect the viability of the KATO III cells and no meaningful proliferative variations were recognized. Moreover, the results obtained from the FCM assay showed that *miR-193a-5p* mimic had no meaningful impact on apoptosis occurrence in KATO III cells (Figure 3B). Rationally, because the KATO III cells are metastatic cell lines, we focused the rest of our study to discover the influences of *miR-193a-5p* mimic of the migration rate of these cells.

### Overexpression of miR-193a-5p inhibited migration of KATO III cell line

**Figure 4:**
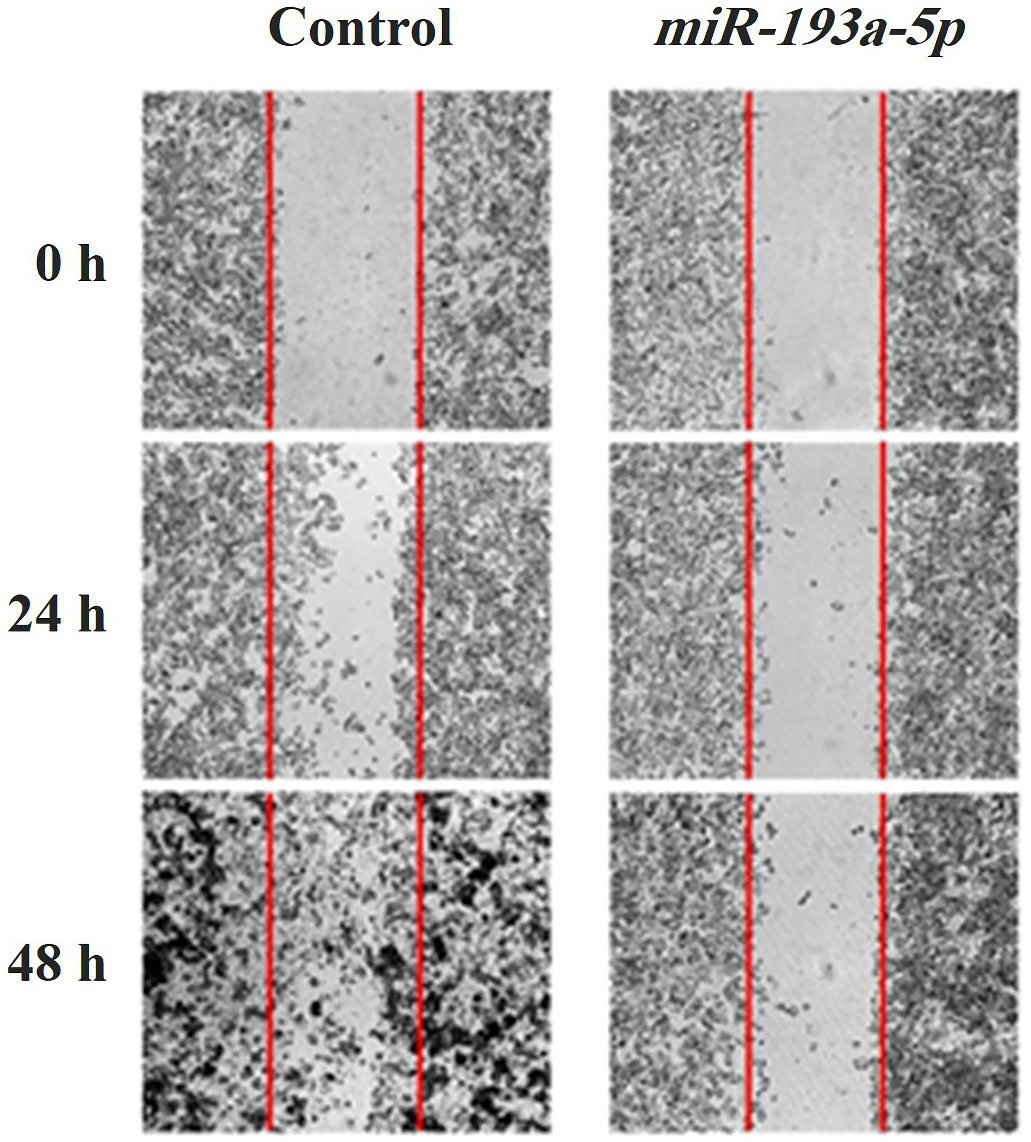
Wound healing assay for evaluation of migration in *miR-193a-5p* transfected KATO III cells has shown that the number of migrated cells in both 24 and 48 h are considerably low in high expressing *miR-193a-5p* cells compared with control-miR transfected cells.

A wound-healing approach was done to evaluate the migration rate of the KATO III cell line in miRNA treated and control-treated groups. The wound space was recorded at 0, 24, and 48 hours. As represented in Figure 4, the transfection of *miR-193a-5p* in KATO III cells, in comparison to the control cells, revealed significant suppression of cell migration in 48 hours. Moreover, for further evaluation of the migration rate of the cells following transfection the genes expression of migratory genes was evaluated to find the impact of *miR-193a-5p* on the migratory ability of the cells.

### Transfection of miR-193a-5p changed metastasis-related genes expression

**Figure 5:**
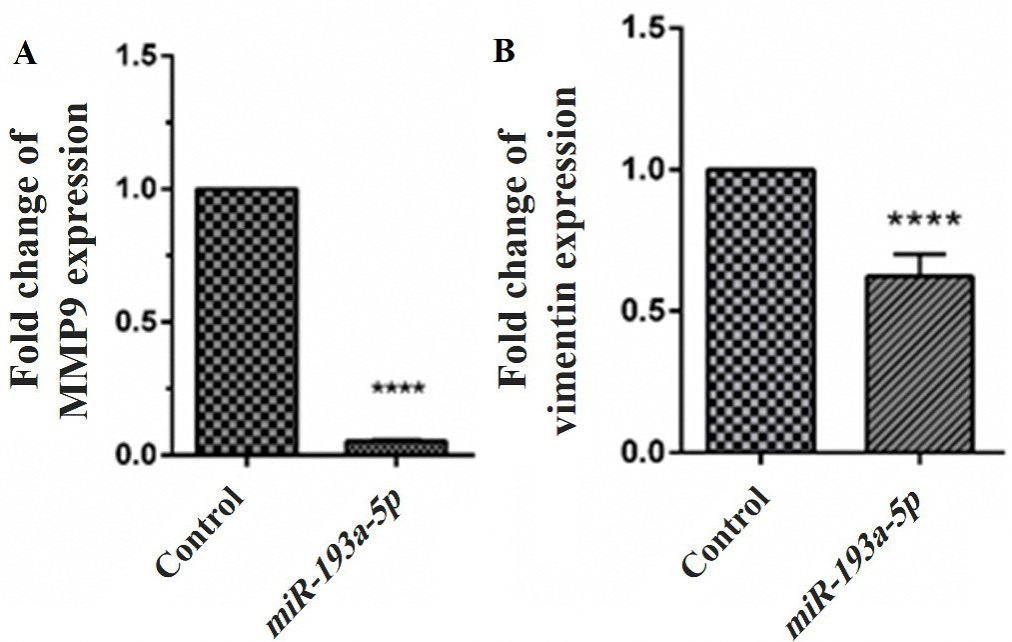
Effect of *miR-193a-5p* mimics transfection on MMP9 (A) and vimentin (B) expression in the mRNA level. Levels of MMP9 and vimentin mRNAs were significantly decreased in cells with induced *miR-193a-5p* expression compared with the control. Cells transfected with a control-miR were considered as control. Data are presented as mean ± SEM (****P < 0.0001).

The effects of *miR-193a-5p* mimic transfection on the mRNA expression of vimentin and MMP-9 as the most important metastatic genes were examined by qRT-PCR assay (Figure 5A, 5B). The findings designated that enhanced expression of *miR-193a-5p* following transfection by its mimic sequences has a significant inhibitory impact on the expression of MMP-9 (*P* < 0.0001) and vimentin (*P* < 0.0001). We could not detect the significant impacts of *miR-193a-5p* mimic transfection on other migratory genes.

## Discussion

As one of the most prevalent causes of mortality from diseases, GC cause a significant global burden of disease to societies. Nowadays, the combination of chemotherapy, radiotherapy and surgery is the common therapeutic method for this malignancy.^8^ However, these treatment strategies have not satisfactory effects against GC in the metastatic phase and fail in many patients in part due to intrinsic or acquired resistance to therapy.^22^ To date, the many of the underlying mechanisms of resistance to chemotherapeutics have been identified, which discussed in more detail elsewhere and are beyond the scope of this manuscript to mention all of them, but the precise mechanism still not fully understood.^9,23^ Among these mechanisms, miRNAs have been identified as one of the pivotal players in GC through posttranscriptional modulation of tumor-related genes.^23^ To date, many miRNAs have been found in different levels of GC pathogenesis ranging from gastritis toward metastatic disease and their functional significance has been proven in numerous studies.^24^ According to the literature, the *miR-193a* family has been published to be disrupted in different kinds of malignancies,^16-19^ but studies on the role of these miRNAs on GC pathways are limited. According to previous studies, *miR-193-5p* has been downregulated in some GC types and its tumor suppressor function is well identified in some experiments.^25,26^ Similarly to these studies, in this experiment, the expression of *miR-193a-5p* was reduced in all investigated three GC cells. However, the KATO III cell line showed the deepest expression of the *miR-193a-5p* level. Therefore, this cell line was chosen for further investigations in this study. In addition, this data is consistent with other bioinformatics data, in which the results showed that the *miRNA-193a-5p* expression is low in various GC cell lines.^27^

As important findings of our study, we determined that *miR-193a-5p* was downregulated in different GC cells. Among these cells, the KATO III cell line, which is a metastatic GC cell line, has the lowest amount of *miR-193a-5p* expression. This preliminary data is in line with previous studies and indicates that *miR-193a-5p* could perform tumor-suppressive functions.^28^ Following the *miR-193a-5p* transfection, we could not recognize significant changes in cell proliferation. The additional confirmation is made via further evaluation of the impact of *miR-193a-5p* on the apoptosis rate of KATO III cells. The apoptosis assay was performed to validate the findings of the MTT assay. The results indicated that *miR-193a-5p* had no significant influence on the apoptosis processes of the cells. On the contrary, overexpression of *miR-193a-5p* could significantly suppress the KATO III cells migration without affecting the cell survival and apoptosis. To investigate the mechanisms of *miR-193a-5p* caused suppression of KATO III cells migration, we investigated the mRNA expression of some metastasis genes, including vimentin, and MMP-9, after miRNA transfection. We estimated that the upregulation of *miR-193a-5p* may regulate the downregulation of vimentin and MMP-9. Therefore, the qRT-PCR analysis revealed that mRNA expression of vimentin and MMP-9 was reduced along with mimic transfection. It is reported that vimentin promotes GC invasion and metastasis through the enhancement of epithelial-mesenchymal transition (EMT)^29,30^ and the concurrent expression vimentin with other cancer-associated genes was observed in numerous cancers.^31^ EMT is the first step in the metastasis of the cancer cells and is defined by the loss of cell-cell adhesion and the receiving of migratory ability and merging evidence suggests that EMT serves as an integral component of GC.^32^

In our study, after the transfection of *miR-193a-5p* to the KATO III cells, we identified the opposite relationship between *miR-193a-5p* and vimentin, modeling that when *miR-193a-5p* is upregulated in cells, the expression of vimentin declines. This observation indicated that *miR-193a-5p* may exert its inhibitory function on the movement of KATO III cells via downregulation of vimentin, which is in line with some other similar studies, which showed that miR-1275 and miRNA-373 reduce the expression of vimentin in GC cells.^33,34^

In addition to vimentin, we evaluated MMP-9 mRNA expression in KATO III cell line, following *miR-193a-5p* mimics transfection and demonstrated that this mimic miRNA decreases the mRNA expression levels of MMP-9. This gene, as one of the members of the MMP metalloproteinase family, is involved in degrading extracellular matrix, thus promoting cancer progression via enhanced migration, angiogenesis, and metastasis.^35^ The higher expression of MMP-9 involves the occurrence, progression, invasion, and metastasis of GC. In addition, this gene can be used as a metastatic predictor and prognostic marker for GC.^36^ In a similar study, increasing miRNA-324 expression leads to MMP-9 reduced expression and inhibited the migration of colorectal cancer cells.^37^ Considering the findings of the current study, it could be assumed that *miR-193a-5p*, maybe by interaction with the 3′-UTR of MMP-9 and vimentin mRNAs regulates other metastasis-associated genes affecting the migration of the cells. In cytotoxicity analysis of* miR-193a-5p* in GC KATO III cells, we could not find significant changes in the viability of the cells. In addition to this, we used flow cytometry assay to evaluate the rate of apoptosis, and consistent with the results from cytotoxicity analysis, *miR-193a-5p*transfection has no statistically significant effects on the apoptosis rate of KATO III cells.

While a few studies reported anti-proliferative and/or pro-apoptotic functions of the *miR-193a-5p*,^28,38^ but in our study, we couldn’t observe such a relationship between *miR-193a-5p* transfection and changes in proliferation or apoptosis indices. This may be due to the fact that, regarding the types of tumors, the different miRNAs cause different effects in tumor cells. One miRNA may be a tumor suppressor in one tumor, but it may be an oncogene in another tumor. These controversies are related to the different signaling pathways influenced by such miRNAs. Therefore, in this study, in addition to the scratch assay, the expression levels of the genes involved in migration were also analyzed.

## Conclusion

Based on the studies and obtained evidence, it is clear that overexpression of *miR-193a-5p* after mimics transfection in KATO III GC cells could significantly harness the movement of the cells. Here, we identified that this miRNA might be included in metastasis of GC cells by regulation of vimentin and MMP-9 genes *in vitro*. More studies for assessment of the underlying signaling cascade and targets of *miR-193a-5p* particularly on animal models or through clinical trials are needed to the potential advantages of applying this therapeutic strategy in GC metastasis therapy.

## Ethical Issues

All experiments and procedures were conducted in compliance with the ethical principles of Tabriz University of Medical Science, Tabriz, Iran and approved by the regional ethical committee for medical research (Ethical code: IR.TBZMED.REC.1397.638).

## Conflict of Interest

The authors have no conflicts of interest to declare.

## Acknowledgments

The authors would like to thank the Immunology Research Center, Tabriz University of Medical Sciences for their support.
